# The Antitumor Effect of Timosaponin A3 through c-Myc Inhibition in Colorectal Cancer Cells and Combined Treatment Effect with 5-FU or Doxorubicin

**DOI:** 10.3390/ijms231911900

**Published:** 2022-10-07

**Authors:** Hyun Min Ko, Wona Jee, Do-il Park, Kwan-Il Kim, Ji Hoon Jung, Hyeung-Jin Jang

**Affiliations:** 1 College of Korean Medicine, Kyung Hee University, Seoul 02447, Korea; 2 Division of Allergy, Immune and Respiratory System, Department of Internal Medicine, College of Korean Medicine, Kyung Hee University, Seoul 02447, Korea

**Keywords:** Timosaponin A3, c-Myc, CNOT2, MID1IP1, HCT116

## Abstract

Timosaponin A3 (TA3), extracted from the rhizome of *Anemarrhena* *asphodeloides* Bunge, has been reported to affect various diseases, such as cancer, Alzheimer’s disease, and allergies. However, the underlying molecular mechanisms and impacts are largely unknown. In the present study, we hypothesized that TA3 induces apoptosis through the inhibition of c-Myc expression via CNOT2 or MID1IP1 in HCT116. An MTT assay and colony formation assay were used to measure cell viability and proliferation. The protein expression of apoptotic markers and oncogenes was measured using immunoblotting and immunofluorescence assays. The interaction between MID1IP1 and c-Myc was confirmed by performing an immunoprecipitation assay. TA3 markedly inhibited colon cancer cell proliferation. Consistently, TA3 regulated the apoptotic proteins pro-PARP and caspase 3. TA3 inhibited the half-life of c-Myc and suppressed its expression in response to serum stimulation. In addition, TA3 enhanced the apoptotic effects of doxorubicin and 5-FU in colon cancer cells. Altogether, our results reveal a mechanism by which TA3 induces apoptosis through inhibiting c-Myc expression via CNOT2 or MID1IP1 in HCT116, which may help in the development of new therapies for colon cancer based on TA3 in the future.

## 1. Introduction

Colorectal cancer is the third most common malignancy worldwide, and diet control, weight management, and exercise can help to reduce the risk for develop colorectal cancer. However, over the past 25 years, morbidity and mortality have increased in adults aged under 50 years worldwide, and approximately 50,000 colorectal cancer patients pass away every year in the United States [1]. Although 5-fluorouracil (5-FU) and doxorubicin (DOX) are mainly prescribed as chemotherapy for colorectal cancer patients, their continuous long-term administration significantly reduces the therapeutic effect due to multidrug and chemical resistance [2,3]. In addition, severe side effects such as lymphopenia and cardiac dysfunction may occur, thus restricting their clinical use. As a result, effective treatment may not be achieved [4,5].

To overcome this issue, many studies have been conducted on relatively low-toxicity, natural product-derived compounds to explore candidate materials to reduce the negative effects while increasing the treatment efficiency of chemotherapies such as 5-FU and DOX. In addition, treatments that inhibit their function by targeting specific molecules such as c-Myc, CCR4-NOT2 (CNOT2), and midline1 interacting protein 1 (MID1IP1), which have recently been identified as oncogenes, have been widely proposed as novel treatments for various cancers, including colorectal cancer [6,7,8]. With this approach, many studies consider that natural products can become lead compounds for molecularly targeted therapeutics [9].

c-Myc, a prototype member of the Myc family, is a transcription factor that is thoroughly controlled in normal cells, while it is overexpressed in human cancer and plays an essential role in cell metabolism, growth, proliferation, and apoptosis [10,11,12]. In addition, c-Myc regulates transcription factors and their signaling pathways in various tumors [13]. Therefore, by regulating the function of c-Myc, one could potentially induce apoptosis and inhibit cancer cell proliferation.

CNOT2 is a subunit of the CCR4-NOT complex that controls mRNA metabolism and the stability of eukaryotic cells and has been reported to play a role in autophagy, apoptosis, proliferation, and angiogenesis in various cancer cells, including those of the colon, lung, and breast [14,15,16,17]. MID1IP1 is a gene that regulates acetyl-CoA carboxylase in the liver and promotes adipogenesis by activating the liver-X receptor; its role as an oncogene has recently been revealed [18]. MID1IP1 is highly expressed in various cancer cells. It has recently been reported that MID1IP1 is present upstream of AMP-activated protein kinase (AMPK) in liver cancer cells and is a negative regulator of AMPK [19]. In addition, it has been reported that the simultaneous knockdown of MID1IP1 and CNOT2 in the liver and in colorectal cancer cells further downregulates c-Myc, thereby contributing to cancer cell growth and apoptosis [8].

*Anemarrhena asphodeloides* Bunge is a herbal medicine used for a long time to treat high fever and diseases such as diabetes [20]. In addition, the anti-cancer activity of *Anemarrhena asphodeloides*, through cell cycle arrest and apoptosis, has been reported in colon, pancreatic, gastric, and liver cancers [21,22,23,24]. Timosaponin A3 (TA3), a steroidal saponin, is a natural substance extracted from the rhizome of *Anemarrhena asphodeloides* Bunge, with effectiveness against various diseases, such as cancer, diabetic osteoporosis, Alzheimer’s disease, allergies, and viral infection [25,26]. However, our knowledge regarding the anti-cancer effects and molecular mechanisms of TA3 in colon cancer remains insufficient.

Our results indicate that TA3 suppresses the expression of c-Myc via downregulation of CNOT2 and MID1IP1 in colorectal cancer cell lines, thereby inducing apoptotic markers. In addition, it was shown that the co-administration of 5-FU or DOX with TA3 has a combined effect that further enhances apoptosis.

## 2. Results

### 2.1. TA3 Inhibits Colon Cancer Cell Viability and Proliferation and Induces Apoptosis

**Figure 1:**
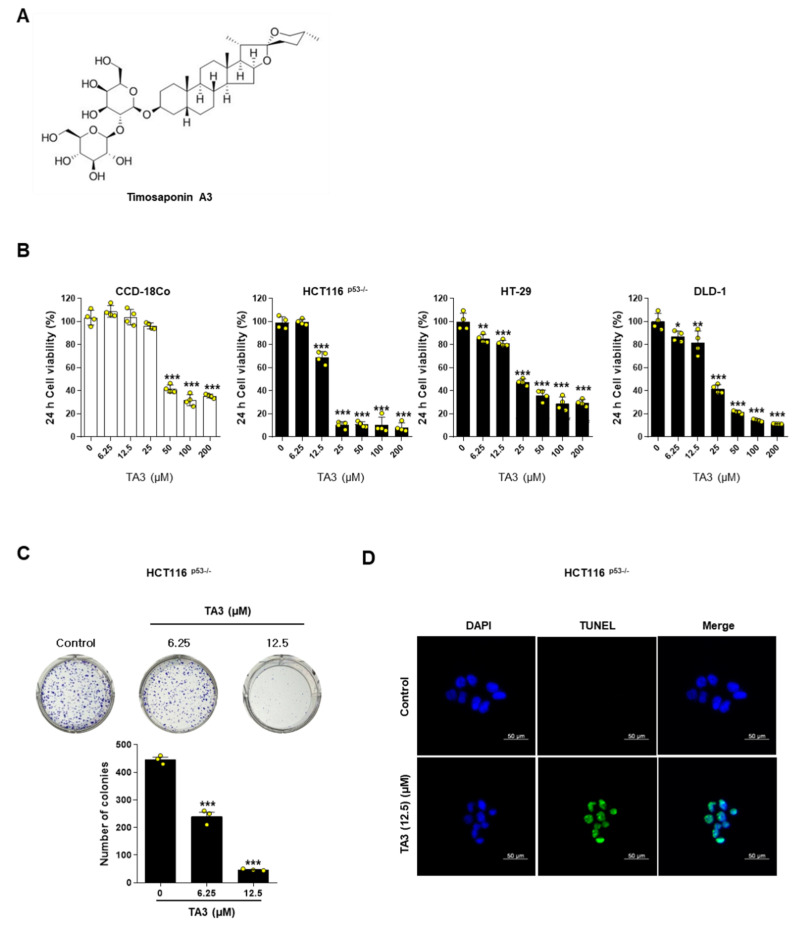
Timosaponin A3 (TA3) inhibits colon cancer cell viability and proliferation and induces apoptosis. (**A**) Chemical structure. (**B**) Effect of TA3 on cell viability in normal colonic cell line CCD-18Co and colorectal cancer cell line HCT116^p53−/−^, HT-29, and DLD-1 cells. Cells were treated with 0, 6.25, 12.5, 25, 50, 100, and 200 μM of TA3 for 24 h. After this, cell viability was evaluated by MTT assay with three independent experiments in each cell. (**C**) Colony formation photos (upper figure) and bar graph (below figure) in HCT116^p53−/−^cells. Cells were treated with 0, 6.25, and 12.5 μM of TA3 for 24 h. HCT116^p53−/−^ cells were treated with TA3 for 24 h and then the new medium was changed for 10 days (every 2–3 days, the new medium was changed). After 10 days, cells were stained with Diff Quick solution 2. Then, cells were dried and counted. Statistical significance was analyzed using one-way analysis of variance (ANOVA) followed by Tukey’s test. *p*-value indicates a significant difference between the groups. Quantification of colonies is shown in the lower panel. * *p* < 0.01, ** *p* < 0.005, *** *p* < 0.001 vs. control. (**D**) HCT116^p53/−^ cell lines treated with TA3 were evaluated for apoptosis activity by TUNEL assay. Images are magnification 400×, scale bar 50 µm.

To check the effect of TA3 on the cell viability and proliferation of various colon cancer cells, MTT, and colony formation assays were performed in colon cancer cells. MTT assay data showed that TA3 inhibited the cancer cell viability dose-dependently in HCT116^p53−/−^, HT-29, and DLD-1 cells (Figure 1B). Interestingly, in CCD-18Co, a non-cancerous colon cell line, when TA3 was administered up to a concentration of 25 uM, cell viability was not affected. Representatively, we performed a colony formation assay in HCT116^p53−/−^ cells, where cell viability was most inhibited when TA3 was administered at a concentration of 12.5 uM. Colonies were inhibited in TA3-treated HCT116^p53−/−^ cells compared with the untreated group (Figure 1C). Additionally, it was confirmed by a TUNEL assay that cell death by TA3 treatment was due to apoptosis in HCT116^p53−/−^ cells (Figure 1D).

### 2.2. TA3 Attenuates the Expression of c-Myc, CNOT2, and MID1IP1 and Induces Apoptosis in Colon Cancer Cells

**Figure 2:**
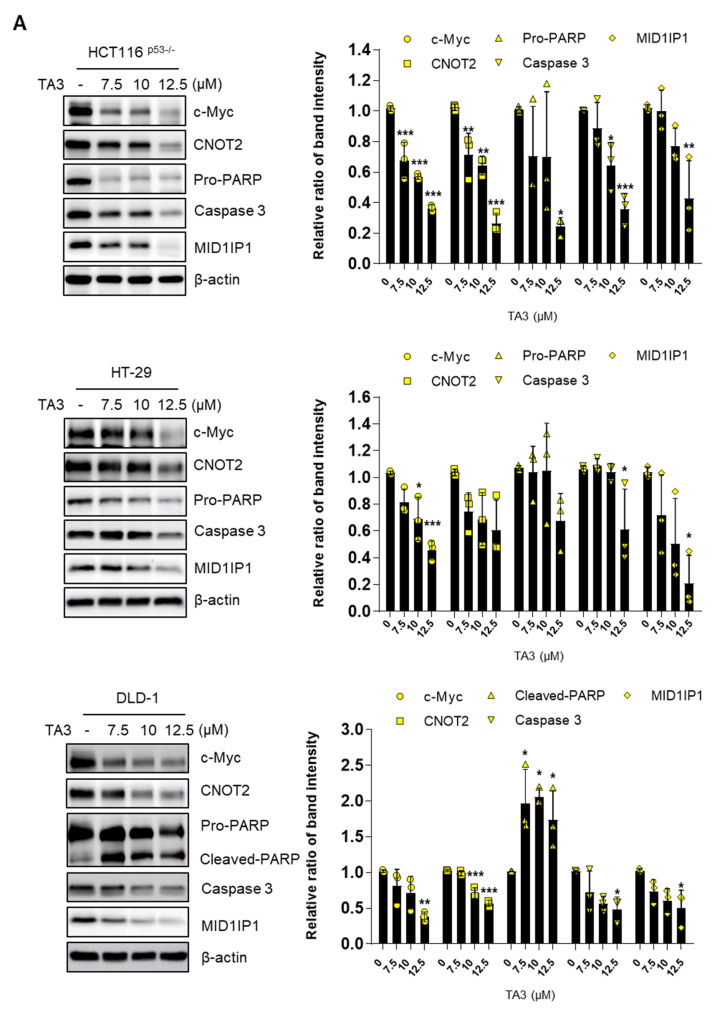

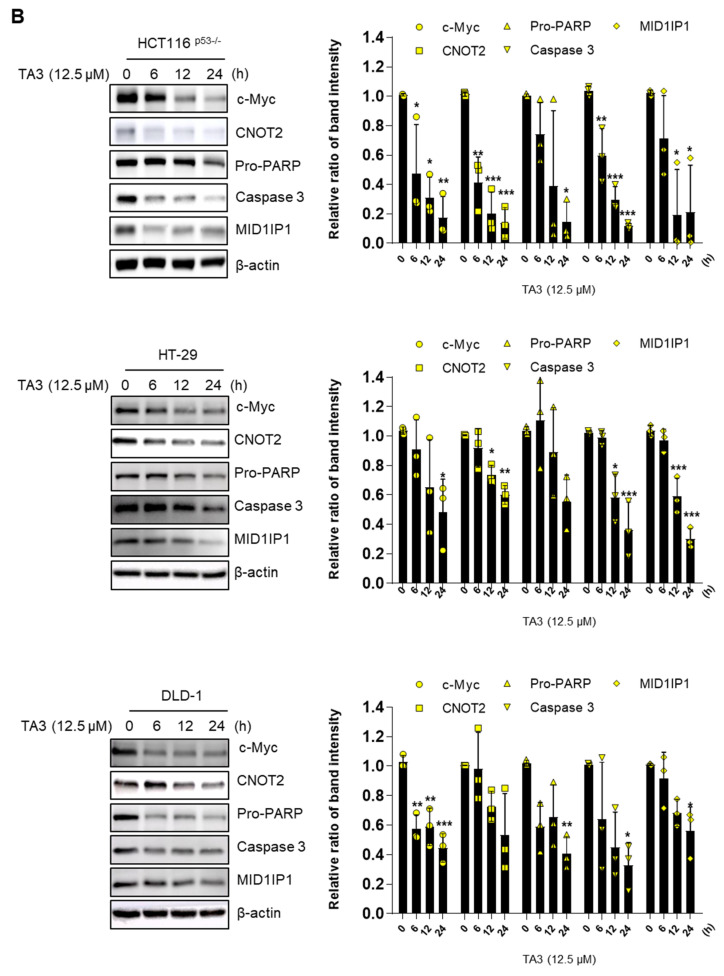
TA3 attenuated the expression of c-Myc in colorectal cancer cells. (**A**) Cells were treated with TA3 (0, 7.5, 10, and 12.5 μM) for 24 h. (**B**) Cells were treated with TA3 (12.5 μM) at various time points for Western blotting (three independent experiments in each cell). Statistical significance was analyzed using one-way analysis of variance (ANOVA) followed by Tukey’s test. *p*-values indicate a significant difference between the groups. Quantification of protein expression is shown in the right panel. * *p* < 0.01, ** *p* < 0.005, *** *p* < 0.001 vs. control.

c-Myc is well known to play a vital role as an oncogene in human cancer cells [27,28]. In addition, c-Myc is involved in cell proliferation, cell cycle, metabolism, and survival in cancer cells [29]. Previously, we showed that the inhibition of CCR4-NOT transcription complex subunit 2 (CNOT2) induces apoptosis through MID1IP1 by activating p53 [17]. Furthermore, previous studies have shown that MID1IP1 regulates cancer cell growth through c-Myc regulation [8]. To check whether TA3 regulates c-Myc, CNOT2, and MID1IP1 expression, a Western blot assay was conducted in colon cancer cells. As shown in Figure 2A,B, TA3 inhibited c-Myc, CNOT2, and MID1IP1 expression in a dose- and time-dependent manner. Furthermore, when cells were treated with TA3, increased apoptosis was confirmed by changes in the expression of apoptotic proteins pro-PARP and caspase 3.

### 2.3. TA3 Inhibits c-Myc Protein Stability

**Figure 3:**
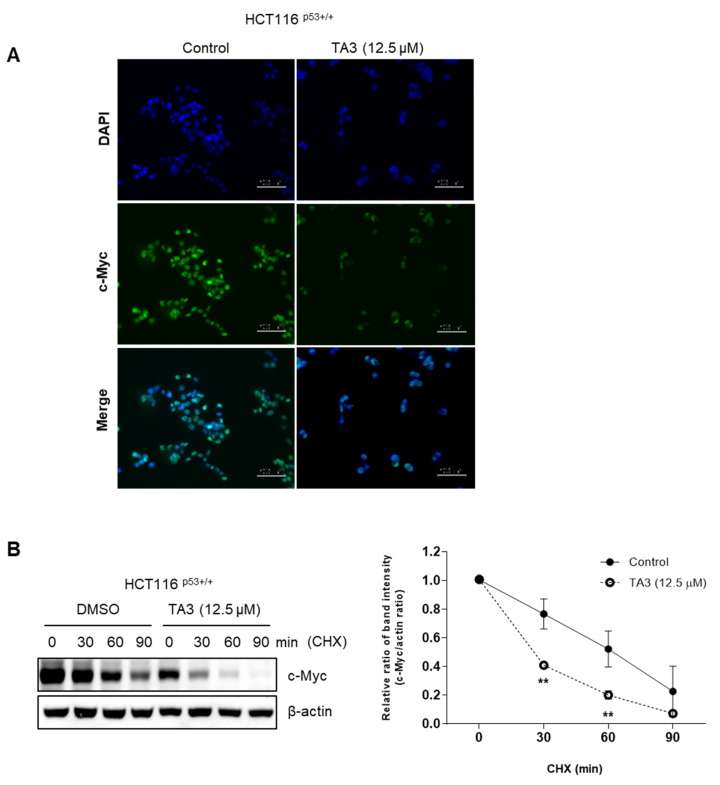
TA3 reduced c-Myc protein stability. (**A**) Cells were treated with TA3 (12.5 μM) for 24 h. Then, cells were fixed with 4% paraformaldehyde and labeled with c-Myc antibody. Images are magnification 200×, scale bar 100 µm. (**B**) Effect of cycloheximide (CHX) on the c-Myc expression in TA3-treated HCT116^p53+/+^ cells. HCT116^p53+/+^ cells were treated with TA3 (12.5 μM) for 24 h, and then exposed to 50 μg/mL CHX at different time points. Statistical significance was analyzed using the unpaired t-test. p-values indicate a significant difference between the groups. Quantification of protein expression is shown in the right panel. ** *p* < 0.005 vs. control.

Next, we checked c-Myc expression using immunofluorescence. As shown in Figure 3A, c-Myc expression was decreased by TA3 treatment. After this, we sought to determine how TA3 regulates c-Myc protein levels. TA3 reduced the stability of c-Myc’s half-life in HCT116^p53+/+^ cells in the presence of cycloheximide compared with the DMSO (control) group (Figure 3B).

### 2.4. TA3 Treatment Decreases Serum-Stimulated Expression of c-Myc

**Figure 4:**
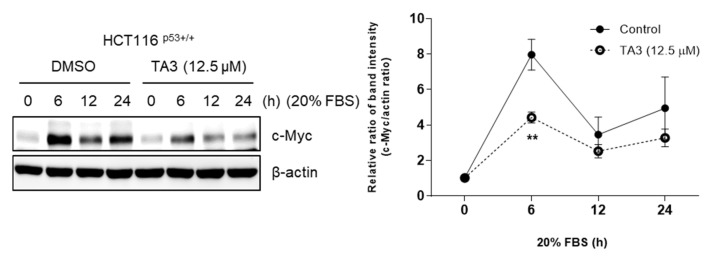
TA3 regulates c-Myc expression by serum stimulation. Cells were treated with TA3 (12.5 μM) and were starved in 0.2% FBS for 24 h and stimulated with 20% FBS for indicated time points. *P*-values indicate significant differences between the groups. Statistical significance was analyzed using the unpaired t-test. Quantification of protein expression is shown in the right panel. ** *p* < 0.005 vs. control.

c-Myc responds quickly to serum stimulation [28]. To confirm whether serum-responsive induction can be affected by TA3, we compared c-Myc expression in HCT116^p53+/+^ cells treated with TA3 or DMSO by serum stimulation (Figure 4).

### 2.5. TA3 Induces Apoptosis with CNOT2 and MID1IP1

**Figure 5:**
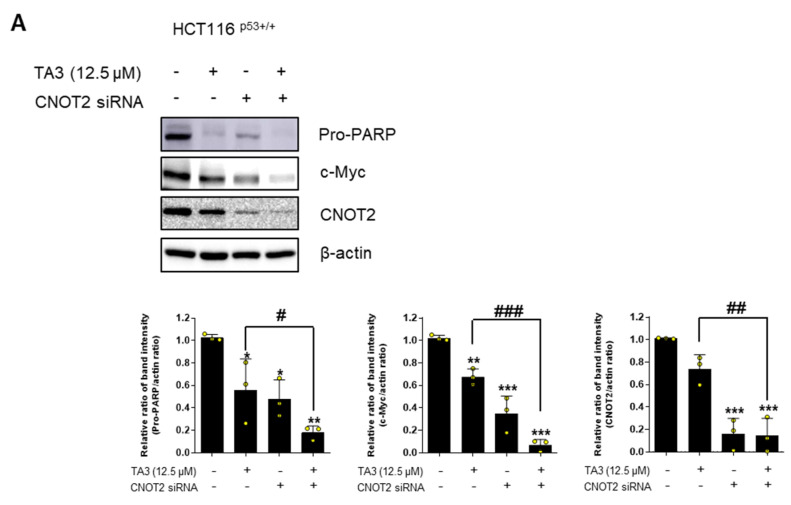

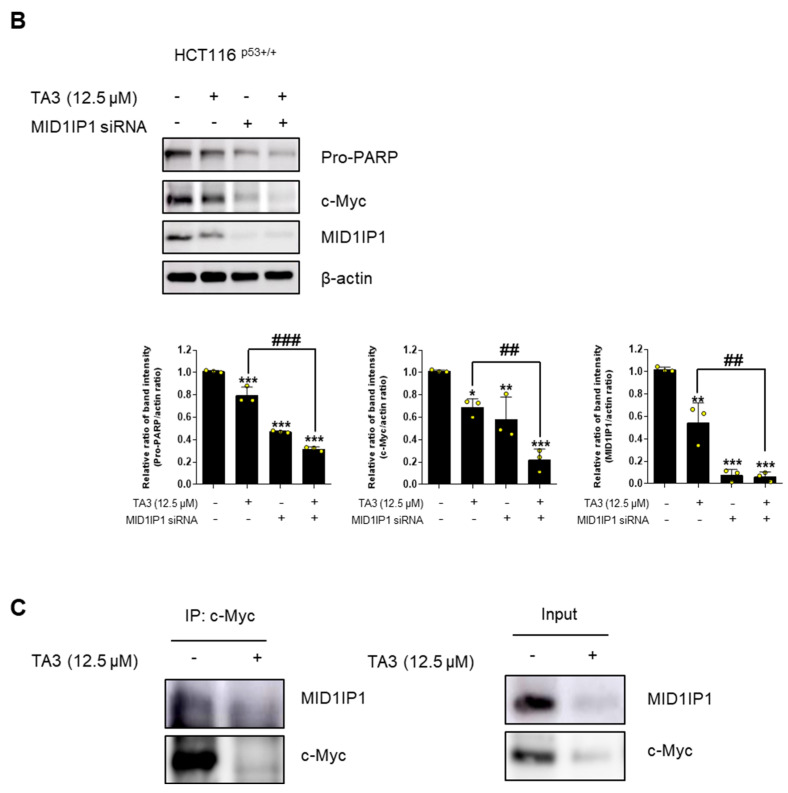
TA3 inhibits c-Myc and induces apoptosis via CNOT2 or MID1IP1. (**A**) HCT116^p53+/+^ cells were transfected with CNOT2 siRNA or control siRNA for 48 h and then cells were treated with TA3 (12.5 μM) for 24 h (three independent experiments in HCT116^p53+/+^ cells). (**B**) HCT116^p53+/+^ cells were transfected with MID1IP1 siRNA or control siRNA for 48 h and then cells were treated with TA3 for 24 h (three independent experiments in HCT116^p53+/+^ cells). Statistical significance was analyzed using one-way analysis of variance (ANOVA) followed by Tukey’s test and the unpaired t-test. *p*-value indicates a significant difference between the groups. Quantification of protein expression is shown in the lower panel. * *p* < 0.01, ** *p* < 0.005, *** *p* < 0.001 vs. control; ^#^ *p* < 0.01, ^##^ *p* < 0.005, ^###^ *p* < 0.001 vs. only TA3-treated group. (**C**) HCT116^p53+/+^ cells were treated with TA3 for 24 h. Then, cells were harvested for co-immunoprecipitation assay.

Previous studies [17] showed that knockdown of CNOT2 induces apoptosis with MID1IP1 in colon cancer cells via p53 activation. Furthermore, MID1IP1 regulates liver cancer growth through c-Myc mediated by CNOT2. These studies led us to believe that TA3 may be related to c-Myc via CNOT2 and MID1IP1. To confirm whether CNOT2 or MID1IP1 plays an essential role in c-Myc regulation via TA3, we knocked down CNOT2 and MID1IP1 with or without TA3 in HCT116^p53+/+^ cells. As shown in Figure 5A, treatment with TA3 reduced pro-PARP and c-Myc expression, and CNOT2 knockdown by siRNA enhanced this reduction in HCT116^p53+/+^ cells. Interestingly, the knockdown of MID1IP1 by siRNA enhanced this reduction (Figure 5B). Furthermore, we tested how TA3 affects the interaction between MID1IP1 and c-Myc through co-immunoprecipitation experiments. As shown in Figure 5C, TA3 inhibited the binding between MID1IP1 and c-Myc.

### 2.6. TA3 Potentiates the Apoptotic Effect with 5-FU or Doxorubicin in Colon Cancer Cells

**Figure 6:**
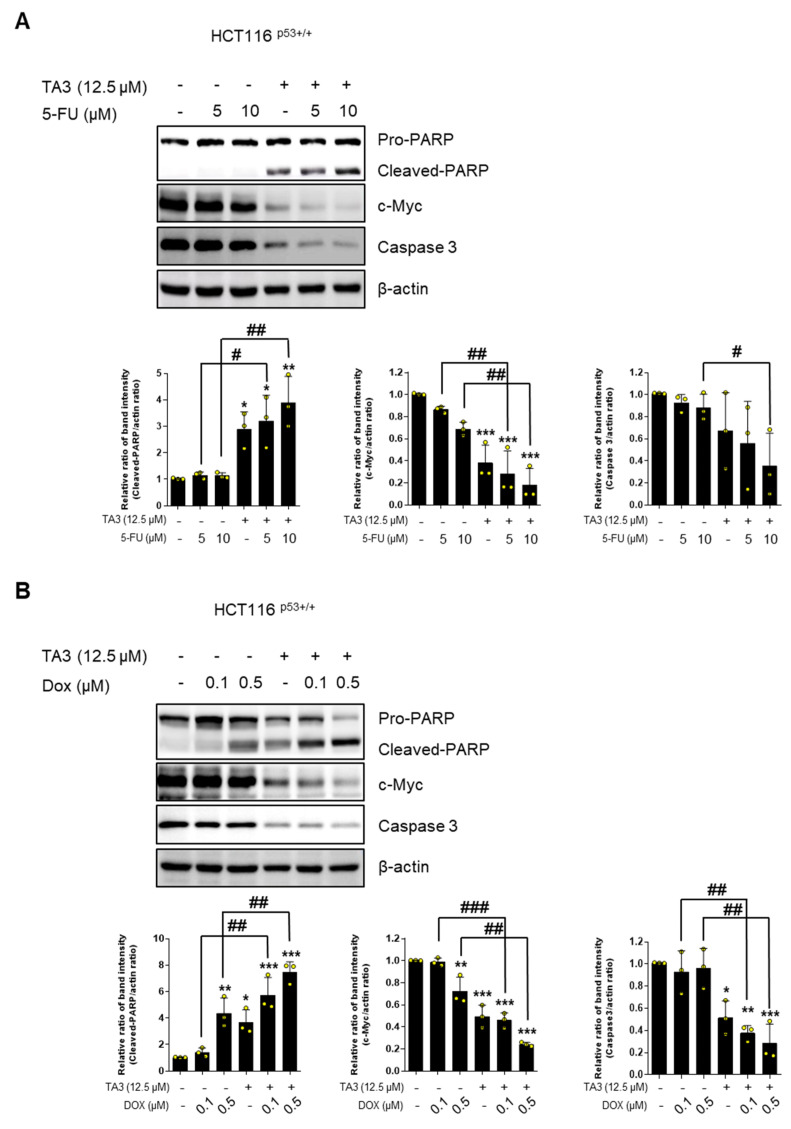
Combination therapy of TA3 with 5-FU or doxorubicin in HCT116^p53+/+^ cells. (**A**) Cells were treated with TA3 (12.5 μM) for 24 h with or without 5-FU. (**B**) Cells were treated with TA3 (12.5 μM) for 24 h with or without doxorubicin. Statistical significance was analyzed using one-way analysis of variance (ANOVA) followed by Tukey’s test and the unpaired t-test. Quantification of protein expression is shown in the right panel. * *p* < 0.01, ** *p* < 0.005, *** *p* < 0.001 vs. control; ^#^ *p* < 0.01, ^##^ *p* < 0.005, ^###^ *p* < 0.001 vs. only 5-FU or DOX-treated group.

It is important to address the issue of the side effects of 5-FU and doxorubicin for cancer therapy. Here, co-treatment with TA3 and 5-FU attenuated the expression of c-Myc and caspase 3 and induced cleaved-PARP compared to TA3 alone (Figure 6A). Co-treatment with TA3 and doxorubicin also attenuated the expression of c-Myc and caspase 3, and induced cleaved-PARP, compared to TA3 alone, in HCT116^p53+/+^ cells (Figure 6B).

## 3. Discussion

Recently, a treatment for colorectal cancer has been developed; however, side effects are still emerging as a problem. Therefore, it is critical to identify new treatments or target mechanisms. Attempting to address this question, our study showed that TA3 is a good candidate for colon cancer treatment.

We previously found that TA3, a steroidal saponin isolated from *Anemarrhena asphodeloides* Bunge, attenuates the suppression of SREBP-1 and induces G2/M cell cycle arrest via the STAT3 and MAPK pathways in pancreatic cancer [22,30]. In addition, TA3 has been reported to exert anti-cancer effects by inhibiting cell migration, invasion, and angiogenesis through various mechanisms in cancers such as lung cancer, liver cancer, breast cancer, renal cancer, and melanoma [31,32,33,34,35,36]. On the other hand, the effect of TA3 in colorectal cancer has been rarely studied. Therefore, to confirm that TA3 negatively regulates c-Myc by activating the tumor suppressor p53, we previously conducted an experiment using two cell lines, HCT116 p53 wild type and null type. We will conduct a mechanism study in cells of TA3 through p53 in the future. Therefore, this study focused on controlling the expression of c-Myc in various colorectal cancer cells, and further, an experiment was conducted focusing only on tumor genes c-Myc, MID1IP1, and CNOT2 using HCT116^p53+/+^. Thus, to the best of our knowledge, this study is the first to reveal that TA3-induced apoptosis through c-Myc requires CNOT2 or MID1IP1 in colon cancer cells.

Before that, we first treated various colorectal cancer cell lines and CCD-18Co, a normal colon cell line, with TA3 by concentration in order to find a concentration of TA3 that has a therapeutic effect on colorectal cancer cells and is not toxic to normal cells. As a result, TA3 has a usable range for treating cancer up to a concentration of 25 μM, and at this concentration, the cell viability of cancer cells is at a level of 15–40%, whereas it has been confirmed that there is no damage to normal colon cells. In addition, in the case of 5-FU, which is used as a treatment for colorectal cancer in actual clinical practice, according to previous studies [37], the IC50 value for the HCT116 cell line was about 14.3 μM, and it did not show cytotoxicity to the normal cell line, CCD-18Co. However, it was confirmed that the cell viability of CCD-18Co decreased to about 60–80% from the 20–50 μM concentration range, and due to this cytotoxicity, it is used within 10 μM concentration when it is mainly used as positive control in anti-cancer studies. Similarly, the flavonoids Apigenin and Luteolin reported as anti-tumor substances had IC50 values of 21.8 and 14.3 μM for HCT116, respectively; however, at a concentration of 40 μM, rapid cytotoxicity to CCD-18Co was observed. Considering these results, it means that the therapeutic range of TA3 against cancer cells is by no means narrow. Therefore, additional experiments were conducted at a concentration of 25 μM that was not toxic to normal cells.

c-Myc is an essential target gene for the development of anti-cancer drugs. c-Myc is overexpressed in various cancer cells, such as breast, colon, and lung cancer [38,39,40,41]. Therefore, the targeting of c-Myc represents a potent chemotherapy [42].

Our previous study showed that CNOT2 is related to MID1IP1 in regulating the expression of c-Myc and inducing apoptosis in cancer cells [8]. Additionally, knockdown of CNOT2 induces p53 expression and apoptosis via MID1IP1 [17]. From previous results, it can be considered that TA3 causes apoptosis through these mechanisms. Based on this information, we found that TA3 induced apoptosis via the TUNEL assay. Indeed, our Western blotting data revealed that TA3 attenuated the expression of pro-PARP and caspase 3, which are related to apoptosis in colon cancer cells. In addition, we found that TA3 inhibited c-Myc expression. Furthermore, our study showed that TA3 reduced c-Myc protein levels, and newly demonstrated that TA3 can regulate the expression of c-Myc rapidly induced by serum stimulation. Interestingly, we found that TA3 inhibited c-Myc through CNOT2 or MID1IP1.

**Figure 7:**
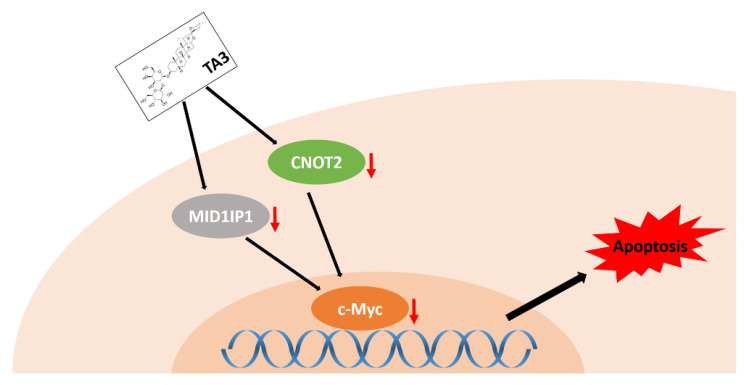
Mechanism of TA3 to induce apoptosis in colorectal cancer by downregulating c-Myc via CNOT2 or MID1IP1.

Our studies reveal a new mechanism for its anti-cancer drug role in inhibiting c-Myc via CNOT2 or MID1IP1 (Figure 7).

However, we suggest that it is necessary to determine further mechanisms regarding the combinational effects of drugs that are already used in clinical trials. Interestingly, TA3 increased its anti-cancer effect with doxorubicin or 5-FU, which are already used to treat colon cancer in the clinical field, as confirmed by Western blotting. These results suggest that TA3 is a new anti-cancer drug candidate for colon cancer cells and it can be administered in combination with existing treatments. Furthermore, we plan to confirm its anti-cancer activity in xenograft mouse models in the future. Moreover, a follow-up study is planned to further investigate the anti-cancer mechanism of TA3 in colorectal cancer in relation to whether TA3 binds directly to CNOT2 and MID1IP1 to interfere with expression.

## 4. Materials and Methods

### 4.1. Chemicals and Antibodies

TA3 (Figure 1A) was purchased from Chemfaces (Wuhan Chemfaces Biochemical Co., Ltd., China). DOX was purchased from Selleckchem (Munich, Germany), while 5-FU was purchased from Sigma-Aldrich (St. Louis, MO, USA). Primary antibodies for CNOT2 (Cat No. 34214) and PARP (Cat No. 9542) were purchased from Cell Signaling Technology (Beverly, MA, USA), MID1IP1 (Cat No.15764-1) was purchased from Proteintech (Rosemont, IL, USA), and c-Myc(Y69) (Cat No. 32072) was purchased from Abcam (Cambridge, UK). Caspase 3 (Cat No. sc-7272), β-actin (Cat No. sc-47778), and secondary antibodies (Cat No. sc-516102; sc-2004) were purchased from Santa Cruz Biotechnology (Dallas, TX, USA).

### 4.2. Cell Culture

HCT116^p53+/+,^ HT-29, and DLD-1 cells were purchased from the American Type Culture Collection (ATCC, Manassas VA, USA) and HCT116^p53−/−^ cells were kindly provided by Dr. Wonchae Choe (Kyung Hee University, Seoul, Korea). Each cell line was cultured in RPMI-1640 containing 1% antibiotics and 10% fetal bovine serum at 5% CO2 and maintained at 37 °C.

### 4.3. Cytotoxicity Assay

The cytotoxicity assay was performed as described in a previous study [17]. HCT116^p53−/−^ cells were distributed in 96-well plates (1 × 10^4^ cells/well), and, the next day, they were treated with TA3 at a concentration range of 0–25 µM for 24 h.

### 4.4. Colony Formation Assay

Colony formation assays were performed using the Diff-Quik kit (Sysmex Corporation, Kobe, Hyogo, Japan), as previously described [43]. Briefly, HCT116^p53−/−^ cells treated with TA3 at a concentration of 0, 6.25, and 12.5 μM were distributed in 6-well plates (1 × 10^3^ cells/well) and cultured for a week to form colonies at 5% CO_2_ and 37 °C. After this, the cells were washed with PBS and fixed for 10 min using Diff-Quik fixation, and the colonies were stained using Diff-Quik solution 2 for 20 min.

### 4.5. TUNEL Assay

To confirm whether TA3 treatment causes apoptosis in HCT116^p53−/−^ cells (1 × 10^5^ cells/well), the DeadEnd™ Fluorometric TUNEL system kit (Promega, Madison, WI, USA) was used. TUNEL-stained cells were visualized using the EVOSR Cell Imaging System (Thermo Fisher Scientific, Waltham, MA, USA).

### 4.6. Western Blot Assay

The drug-treated cells (2 × 10^5^ cells/well) were lysed using a lysis buffer (Cell Signaling Technology, Beverly, MA, USA). Cell lysates were separated by SDS-PAGE (8–12%) and transferred to nitrocellulose membranes. Subsequently, the immunoblotting process was implemented according to a previous study [44]. The dilution ratio of the antibody used was as follows: c-Myc (1:1000), CNOT2 (1:1000), MID1IP1 (1:1000), PARP (1:1000), caspase 3 (1:1000), β-actin (1:5000), goat anti-mouse IgG-HRP (1:5000), and goat anti-rabbit IgG-HRP (1:5000). Experiments were performed at least three times, and representative images were selected and posted.

### 4.7. Immunofluorescence Assay

HCT116^p53+/+^ cells (1 × 10^5^ cells/well) treated with or without TA3 were fixed with 4% paraformaldehyde for 20 min and at room temperature for 10 min, permeabilized with 0.1% Triton X-100, and blocked with 3% BSA for 1 h. Cells were then labeled with a specific antibody against c-Myc (1:100) at 4 °C overnight. Subsequently, the cells were incubated with Alexa Fluor 488 goat anti-rabbit IgG antibody (1:500) (Invitrogen, Waltham, MA, USA) at 4 °C for 2 h. The experiment was performed according to a previous study [45].

### 4.8. RNA Interference

Control, MID1IP1, and CNOT2 siRNAs were purchased from Bioneer (Daejeon, Korea), and RNA interference was performed as described previously [8]. Briefly, HCT116^p53+/+^ cells were distributed in a 6-well plate (7 × 10^4^ cells/well) and cultured overnight. The next day, transfection was performed with control, MID1IP1, and CNOT2 siRNA using INTERFERin^®^ (Poly-plus-transfection SA, Illkirch, France) transfection reagent, according to the manufacturer’s protocol.

### 4.9. c-Myc Stability Using Cycloheximide Chase Assay

The cycloheximide chase assay was described in a previous study [46]. HCT116^p53+/+^ cells (2 × 10^5^ cells/well) were exposed to TA3 for 24 h and then treated with the protein synthesis inhibitor cycloheximide at a concentration of 50 µg/mL for 0, 30, 60, and 90 min. Western blotting was performed to confirm the expression levels of c-Myc and β-actin.

### 4.10. Induction of c-Myc by Serum Stimulation

After seeding HCT116^p53+/+^ cells in 6-well plates (2 × 10^5^ cells/well), the cells were starved in serum-free medium for 24 h. Subsequently, TA3 diluted in a medium containing 20% FBS was incubated at a concentration of 12.5 µM for 0, 6, 12, and 24 h and then the cells were harvested using a cell scraper.

### 4.11. Immunoprecipitation Assay

Immunoprecipitation was performed as previously described^30^. Briefly, TA3-treated or untreated HCT116^p53+/+^ cells (2 × 10^5^ cells/well) were lysed in lysis buffer. Cell lysates were then incubated overnight with MID1IP1 antibody. The next day, Protein A or G beads (Santa Cruz Biotechnology, Santa Cruz, CA, USA) were added and incubated on a rotator at 4 °C for 5 h, and then washed three times with lysis buffer and boiled. Finally, the bound protein was subjected to Western blot analysis.

### 4.12. Statistical Analysis

All experiments were performed with at least three individual repetitions. The significance of each comparison was analyzed by one-way analysis of variance (ANOVA) followed by Tukey’s test using GraphPad Prism software (Version 5.0, San Diego, CA, USA), and comparisons between the two groups were analyzed by an unpaired t-test. All experimental data are expressed as the mean ± standard deviation.

## 5. Conclusions

Our new findings provide further insights into whether the inhibition of c-Myc expression by TA3 might involve cross-talk with the ribosomal proteins or respond to yet-unidentified signals. In addition, the relationship between TA3 and c-Myc can be better understood by creating a biological model system. In conclusion, our findings could be helpful for the discovery of anti-colon cancer drugs in the future.

## Data Availability

Not applicable.
